# Analectic

**Published:** 1830

**Authors:** 


### MISCELLANEOUS INTELLIGENCE.

*ANALECTIC, ANALYTICAL, AND ORIGINAL.*

### I. ANALECTIC.

### Selected Review.

*Of some Symptoms in Children erroneously attributed to Congestion of the Brain.* By Robert Gooch, M. D.

The above is the title of the sixth chapter of Dr. Gooch's recent publication, and the subject is of very great importance. The chapter opens in a ludicrous manner, considering the gravity of the subject; but the moral inculcated deserves to be remembered.

"I remember when a boy reading a story of two knights-errant who arrived on the opposite sides of a pedestal surmounted by a shield; one declared it was gold, the other that it was silver; growing angry, they proceeded to blows, and after a long fight each was thrown on the opposite side of the shield to that where he began the fight—when both immediately detected their error: the knight who had said it was silver finding that on the opposite side it was gold, and the knight who had said it was gold finding that on the opposite side it was silver. This story, a little modified, is a good illustration of the state of medical opinion in this age, perhaps in all ages; medical men have no occasion to tilt, for they all throng on one and the same side of the shield; they look only at the golden side, and never dream of the possibility that on the opposite side it may be of a different metal." 355.

Dr. G. properly remarks that two sets of symptoms are distinguishable in cases of disease, and require to be discriminated. One set of symptoms forms what may be called the physiognomy of the complaint—the other indicates the morbid state of organization on which the disease depends.

"Two patients complain occasionally of dimness of sight, swimming of the head, singing in the ears, and observe that if they turn the head on one side to look at an object they feel as if they should fall; but the one is plump, florid, and has a full pulse; the other is pale and thin, has cold hands and feet, and a pulse small and feeble. One practitioner bleeds them both; the other bleeds the one, but does all he can to give blood to the other. The latter cures both his patients; the former cures the one but ruins the health of the other; but such is the nature of the human mind that the cases *for* a preconceived opinion are retained easier than those *against* it. He remembers his good deed, forgets the other, or calls the case 'anomalous,' and marches on, without the slightest doubt that bleeding is the universal and sovereign remedy for dimness of sight, swimming of the head, and singing in the ears, save and except only in 'anomalous' cases." 357.

Dr. G. is anxious to call the attention of medical men to a disorder of children which he finds invariably attributed to, and treated as, CONGESTION OR INFLAMMATION of the brain; but which he is convinced often depends on, or is connected with, an opposite condition of the circulation. It is chiefly indicated by heaviness of head and drowsiness. The age of the little patients is generally from a few months to two years or three. They have, in our author's experience, been rather small of their age, in delicate health, and exposed to debilitating causes.

"The physician finds the child lying on its nurse's lap, unable or unwilling to raise its head, half asleep, one moment opening its eyes, and the next closing them again with a remarkable expression of languor. The tongue is slightly white, the skin is not hot, at times the nurse remarks that it is colder than natural; in some cases there is at times a slight and transient flush; the bowels I have always seen already disturbed by purgatives, so that I can scarcely say what they are when left to themselves; thus the state which I am describing is marked by heaviness of the head and drowsiness, without any signs of pain, great languor, and a total absence of all active febrile symptoms. The cases which I have seen have been invariably attributed to congestion of the brain, and the remedies employed have been leeches and cold lotions to the head, and purgatives, especially calomel. Under this treatment they have gradually become worse, the languor has increased, the deficiency of heat has become greater and more permanent, the pulse quicker and weaker, and at the end of a few days or a week, or sometimes longer, the little patients have died with symptoms apparently of exhaustion. In two cases, however, I have seen, during the last few hours, symptoms of oppressed brain, as coma, stertorous breathing, and dilated and motionless pupil." 358.

Dr. Gooch relates an instructive case illustrative of the above remarks, where leeches were applied to a child two years old, and the result was fatal. This case and some incidental remarks are so interesting that we shall here insert them.

"A little girl about two years old, small of her age and very delicate, was taken ill with the symptoms which I have above described. She lay dozing, languid with a cool skin, and a pulse rather weak, but not much quicker than natural. She had no disposition to take nourishment. Her sister having died only a week before of an illness which began exactly in the same way, and which was treated by leeches and purgatives ; and some doubts having been entertained by the medical attendant of the propriety of the treatment, leeches were withheld, but the child not being better at the end of two days, the parents, naturally anxious about their only surviving child, consulted another practitioner. The case was immediately decided to be one of cerebral congestion, and three leeches were ordered to be applied to the head. As the nurse was going to apply them, and during the absence of the medical attendants, a friend called in who had been educated for physic, but had never practised it, and who had great influence with the family : he saw the child, said that the doctors were not sufficiently active, and advised the number of leeches to be doubled. Six, therefore, were applied ; they bled copiously ; but when the medical attendants assembled in the evening they found the aspect of the case totally altered, and that for the worse : the child was deadly pale, it had scarcely any pulse, its skin was cold, the pupils were dilated and motionless when light was allowed to fall on them, and when a watch was held to its eyes it seemed not to see ; there was no squinting. Did this state of vision depend on the pressure of a fluid effused into the brain since the bleeding, and during this exhausted and feeble state of circulation, or did it depend on the circulation of the brain being too languid to support the sensibility of the retina ? It is well known that large losses of blood enfeeble vision. I saw a striking instance of this in a lady who flooded to death. When I entered the chamber she had no pulse, and she was tossing about in that restless state which is so fatal a sign in these terrific cases. She could still speak, asked whether I was come, (she knew I had been sent for,) and said 'am I in any danger ?—How dark the room is !—I can't see.' The shutters were open, the blinds up, and the light from the window facing the bed fell strong on her face. I had the curiosity to lift the lid, and observe the state of the eye ; the pupil was completely dilated, and perfectly motionless, though the light fell strong on it. Who can doubt that here the insensibility of the retina depended on the deficiency of the circulation ? But to return to the little patient. The next day she had vomited her food several times ; it was therefore directed that she should take no other nutriment than a desert spoon-ful of ass's milk every hour, and this was strictly obeyed, and continued for several days. The child wasted, her features grew sharp, every now and then she looked fretful, and uttered a faint squeaking cry; the eye-balls became sunk in the socket, like those of a corpse that had been dead a month; the skin continued cool, and often cold, and the pulse weak, tremulous, and sometimes scarcely to be felt. Under this regimen, and in this way, she continued to go on for several days. At times she revived a little, so as to induce those who prescribed this treatment to believe confidently that she would recover, and she clearly regained her sight, for if a watch was held up to her she would follow it with her eyes. She lived longer than I expected; a full week, and then died with the symptoms of exhaustion, not with those of oppressed brain. The head was opened by a surgeon accustomed to anatomical examinations, and nothing was found but a little more serum than is usual in the ventricles." 361.

Our author concludes that the heaviness of the head and drowsiness, which were attributed to congestion in the brain, really depended on a deficiency of nervous energy—that the bleeding and scanty diet aggravated this state, "and insured the death of the child."

Dr. G. was summoned one day to see a child, to whose head the medical practitioner was about to apply leeches. He found the child lying on its nurse's lap, exactly in the state already described; with the same unwillingness to hold up its head—the same drowsiness—languor, absence of heat and of all symptoms of fever. Dr. G. took the medical attendant into another room, and told him the history of the preceding case and of several others of a similar kind. Instead of applying the leeches, they agreed to give from a pint and a half to a quart of ass's milk in the 24 hours—and to take ten minims of the aromatic spirit of ammonia every four hours. When they met next day, the nurse was walking about the nursery with the child upright in her arms. It looked happy and was laughing. The same plan was continued another day, and on the succeeding day Dr. Gooch took his leave.

"So inveterate is the disposition to attribute drowsiness in children to congestion of the brain, and to treat it so, that I have seen an infant, four months old, half dead from the diarrhœa produced by artificial food, and capable of being saved only by cordials, aromatics, and a breast of milk; but because it lay dozing on its nurse's lap two leeches had been put on the temples, and this by a practitioner of more than average sense and knowledge. I took off the leeches, stopped the bleeding of the bites, and attempted nothing but to restrain the diarrhœa and get in plenty of nature's nutriment, and as I succeeded in this, the drowsiness went off, and the child revived. If it could have reasoned and spoken it would have told this practitioner how wrong he was; any one, who from long defect in the organs of nutrition, is reduced so that he has neither flesh on his body, nor blood in his veins, well knows what it is to lay down his head and doze away half the day without any congestion or inflammation of his brain. This error, although I have specified it only in a particular complaint of children, may be observed in our notions and treatment of other diseases, and at other periods of life If a woman has a profuse hæmorrhage after delivery, she will probably have a distressing head-ache, with throbbing in the head, noises in the ears, a colourless complexion, and a quick, weak, often-thrilling pulse, all which symptoms are greatly increased by any exertion. I have seen this state treated in various ways, by small opiates, gentle aperients, and unstimulating nourishment, with no relief. I have seen blood taken away from the head, and it has afforded relief for a few hours, but then the head-ache, throbbing and noises have returned worse than ever; the truth is, that this is the acute state of what in a minor degree and in a more chronic form occurs in chlorosis, by which I mean pale-faced amenorrhœa, whether at puberty or in after life. It may be called *acute chlorosis,* and like that disease is best cured by steel, given at first in small doses, gradually increased, merely obviating constipation by aloetic aperients." 365.

Dr. Gooch is unwilling to encumber this paper with a multiplicity of cases, but states generally that the above are only specimens of a class, of which he has seen enough to convince him that they deserve the attention of the profession. Dr. Gooch refers to the excellent remarks of Dr. Marshall Hall on this class of affections. Dr. G. candidly acknowledges that he has been anticipated by Dr. Hall on this point, but so far from regretting it, he rejoices to find himself supported by such good authority.

"The children who were the subjects of this affection, and were thus treated, died not with symptoms of oppressed brain, but with those of exhaustion, and on examining the head after death, the blood vessels were unusually empty, and the fluid in the ventricles rather in excess; in two instances death was preceded by symptoms of effusion, &c. blindness, a dilated pupil, coma, and convulsions; and after death the ventricles were found distended with fluid to the amount of several ounces, the sinuses, and veins of the brain being remarkably empty. I believe the prevalent notion of the profession is, that all sudden effusions of water into the brain are the result of inflammatory action ; but putting aside for a moment this dogma of the schools, consider the circumstances of this case. For several days before death, all that part of the circulating system which was cognizable to the senses, was at the lowest ebb, consistent with life, and after death the blood-vessels of the brain were found remarkably empty of blood, and the ventricles unusually full of water. From such facts I can draw no other inference than this, that this sudden effusion was a passive exudation from the exhalents of the ventricles occasioned by a state of the circulation the very opposite to congestion or inflammation. This is corroborated by the dissection of animals which have been bled to death. Drs. Saunders and Seeds, of Edinburgh, found that in animals bled to death, whether from veins or arteries, there was found more or less of serous effusion within the head and Dr. Kelly thus expresses himself :—'If instead of bleeding usque ad mortem we were to bleed animals more sparingly and repeatedly, I have no doubt that we should succeed in draining the brain of a much larger quantity of its red blood ; but in such experiments we shall, I think, find a larger effusion of serum." * * * 'Though we cannot, by general depletion, entirely or nearly empty the vascular system of the brain as we can the vessels of the other parts of the body, it is yet possible, by profuse hæmorrhages to drain it of a sensible portion of its red blood, that the place of this spoliation seems to be supplied both by extra and intra vascular serum, and that watery effusion within the head is a pretty constant concomitant or consequence of great sanguineous depletion.' But if this is true, it is of great practical importance, for if we take delicate feeble children, and by bleeding and purging for an imaginary conjestion of the brain, reduce their circulation to a very low ebb and keep it so, we run the risk of producing that very effusion of serum into the brain which we are endeavouring by our remedies to prevent." 368.

Dr. Gooch does not expect that medical men will take his word as conclusive evidence for the truth of this paper—nor does he wish it. All he asks is that they will allow his observations and reasonings to lead them to look out for similar cases, and judge for themselves.

"With regard to the point that heaviness of head and drowsiness in children often depend not on congestion, but on deficiency of nervous power, and require for their cure not depletion, but support, I am quite satisfied that candid observers will find that I am right. With regard to the other point, that sudden effusion of serum may take place in the brain from a state of the circulation, the opposite to congestion or inflammation, it is more likely, even if true, to be overlooked ; for such is the force of preconceived opinion, and such the prevalent notions on the subject, that the following will be the process in most minds. A child has been suffering some obscure symptoms for many days, when suddenly and unexpectedly it becomes blind, its pupils are dilated and motionless, it becomes convulsed, comatose, and dies. On opening the head serum is found in the ventricles, and without any further enquiry it is immediately taken for granted, that this effusion was the effect of overlooked inflammation of the brain, and regret is felt that active depletion had not been employed ; the inference may be a correct one ; all I contend for is that it should not be taken for granted, but that those circumstances should be minutely enquired into which throw light on the state of the circulation in which the effusion occurred." 374.

We strongly recommend Dr. Gooch's observations to the attentive consideration of our professional brethren.

*Medico-Chiurgical Review.*

---

*On the Inefficacy of Ammonia and the Efficacy of the Cold Affusion in the Treatment of Poisoning with Hydrocyanic Acid.*—Some years ago, Mr. Murray, of London, proposed ammonia as an antidote for poisoning with prussic acid, and the trials he made with it on the lower animals were so satisfactory, that he advertised his willingness to take a dose sufficient to cause death in ordinary circumstances, provided a competent person should be at hand to administer his antidote. The investigation, of which we are now to give an account, will probably satisfy him that he might have found reason to repent his rashness, had any one been so curious as to accept his offer.

Dr. Herbst, after repeated experiments, has come to the conclusion, that ammonia, even in a concentrated state, when administered to an animal which has taken a dose of prussic acid large enough to prove fatal, and when not administered till the symptoms have existed for some space of time, will hardly ever save its life. But he admits, that in cases in which the dose has not been adequate to occasion death, yet causes severe symptoms, this remedy is of substantial benefit, by mitigating and shortening its effects. He further objects to its general use as antidote, that in a tolerably concentrated state, it instantly excoriates the parts to which it is applied, and that in a state so dilute as to be free from this inconvenience its efficacy ceases. Having detailed two cases in which large and repeated doses of ammonia gave but temporary relief, and failed to counteract the fatal effects of the poison, he relates several calculated to establish the superior efficacy and certainty of the cold affusion.

"When the dose of the poison was insufficient to prove fatal, two affusions were commonly sufficient to dispel every unpleasant symptom. When the dose was larger, it was requisite to repeat it more frequently, and to persevere for a longer time. The certainty of success depended greatly on the early employment of the remedy. It was most to be trusted when resorted to immediately after the administration of the poison, or at all events during the convulsive stage, while the muscles were in a state of contraction, the eyes staring, insensible, and immoveable, the head drawn backwards, and the extremities extended. This stage is rapidly succeeded by general relaxation of the body, gradually increasing slowness and feebleness of the respiration, a slow, weak, or almost imperceptible pulse ; and death is then close at hand. Even in this paralytic condition, the cold affusion will sometimes recal the expiring spark of life ; then the convulsions of the muscles were renewed, the extremities became again hard and immoveable, and, by the gradual abatement of the spasms, the effects of the poison were slowly dispelled."

1. A full grown spaniel of a small breed got six grains of Schrœder's acid. Immediately he uttered a piteous howl, fell down, stretched out his legs, and in a few seconds appeared dead. Cold water was then poured over its head and back, and subsequently over the whole body ; when the breathing began to return, the animal in a few minutes shook itself, and then tried to rise. In a quarter of an hour he was able to stagger forward a little. In an hour he had recovered entirely. 2. A strong pointer bitch, five years old, took eight drops of Ittner's prussic acid. In a few minutes she staggered, drew her head backwards, and was on the point of falling, when cold water was dashed over her head. Instantly she recovered, and in a few seconds she was as lively as before the experiment. Several fresh small doses of the acid were subsequently given, and always with the effect of developing its narcotic action ; but they were as uniformly removed by a fresh affusion. In fifteen minutes, violent vomiting of tough white mucus supervened, and she slavered much. These symptoms, however, also ceased in an hour and a half, when she took her customary meal. 3. Next morning eight grains were again administered to the same animal. Immediately she tumbled down, and was attacked with opisthotonos of the most violent kind, the breathing became more and more obstructed and feeble, and speedy death was evidently threatened. No time was now lost in applying the cold affusion. In a single minute the breathing became regular, she raised her head, looked bewildered around her, but continued lying. After a second affusion she stood up, walked, but with difficulty, and in an hour was so well that no sign of the action of the poison could be observed. 4. The most decisive trial was the following. Two young poodles were chosen, of the same age and size, and accustomed to the same sort of food. To one was given four drops of Ittner's acid, which had the effect only of causing temporary staggering. Eight drops were then given, and caused loud cries, mucous vomiting, and opisthotonos. From this dose too he was beginning to recover, when four drops more were given ; and death then ensued four minutes after the first administration of the acid. The total quantity of acid amounted to seven grains. This quantity was next given to the other dog at one dose. He immediately turned round, staggered, and fell down, incapable of stirring, and insensible. The head was drawn strongly backwards, the legs were extended, and in scarcely half a minute afterwards, the breathing was imperceptible, and the pulse hardly to be felt. The cold affusion of the head was then resorted to without delay, as the muscles were flaccid, and the animal appeared dead. At first it had no effect. The earliest sign of returning animation was renewal of the opisthotonos, and extension of the flaccid extremities ; at the same time he uttered a feeble cry, which soon afterwards became stronger. The stiffness of the body continued a long time. The affusion was then repeated over the whole body. The crying continued, and touching the body seemed to cause pain. The affusion being repeated every fifteen minutes, the respiration at length became somewhat stronger ; but it always returned to its feeble state in the intervals. At eight in the evening, (the author does not state the interval after the beginning of the experiment,) the animal was completely restored to health, ran about, barked, and took his food as if he had never been ill. 5. Nine drops of Schrœder's acid were injected into the external jugular vein of a little house dog, six months old, in five minutes, twelve drops more, and other five minutes, fifteen drops, without any distinct effect, except quickness, weakness, and irregularity of the action of the heart. Twelve grains (27 drops) were then injected at once, upon which there ensued involuntary discharge of fœces, most violent tetanus, and all the customary symptoms, followed by flaccidity of the body and almost imperceptible breathing. The first cold affusions made the animal spring high up, and when continued, they removed in no long time the paralysis of the hind legs, which was the last symptom that remained. In three quarters of an hour he had recovered completely. 6. Three days afterwards fifty drops were injected into the opposite jugular vein of the same animal. The tetanus was even more violent than before. The cold affusion was not resorted to, until the whole body was in a state of paralytic flaccidity, the muscles of the eyes alone preserving their spasmodic contraction. The effect of the remedy was equally remarkable as in any former experiment or after the first application, the breathing immediately became stronger ; and in four hours the dog was as lively as ever. The same beneficial results were invariably obtained in other experiments, in which the poison was applied to the eye, to the inner membrane of the nose, or to external wounds. When the dose was so large that its effects were beyond the reach of the cold affusion, it was observed to prove fatal instantaneously, that is, almost before there was time to remove the remedy.—*Archiv fur Anatomie and Phosiologie, No.* 2, 1828. *New-York Med. Phys. Journal.*

---

### 3. Christison on Poisons.

*Evidence of General Poisoning.*—The evidence of the existence or non-existence of poisoning in general (that is, without reference to any particular poison) is derived from five sources—

the symptoms,—the post-mortem appearances,—chemical analysis,—experiments on animals—moral circumstances. It will not be uninteresting or useless to [illegible]uch a little on each of these subjects, especially as they have been but imperfectly investigated by preceding writers.

*Evidence from symptoms.*—Not many years ago questions of poisoning were decided by symptoms alone. But no such evidence is now trusted to, per se. Still the symptoms are great auxiliaries, and in some cases, as of mineral acids, arsenic, sublimate, and strychnine, they are almost conclusive. The chief characteristics of the symptoms in poisoning are that they commence suddenly, proceed rapidly t[illegible] a fatal issue—that they increase steadily—are uniform throughout their course—begin after a meal—and appear when the body is in a state of health. The exceptions to each of these characteristics are very numerous, and so very evident that we shall not dwell on them. Thus the first characteristic is exceedingly liable to exceptions, where the dose is small. Arsenic, digitalis, strychnine, &c. may be introduced into the system slowly, and yet so as to produce their specific and fatal effects at last. With respect to the appearance of symptoms of poisoning "*very soon after a meal*," we know that this is the time at which some of the most fatal disorders commence, as apoplexy, cholera, perforation of the stomach from chronic ulceration, &c. At the same time it is to be recollected that the diseases which commence immediately after food, and proceed to a fatal and sudden termination, are few in number, and none of them by any means frequent. The occurrence of such a circumstance may therefore, justly be deemed suspicious. The same may be said respecting the other items above enumerated. The following passage is curious and interesting.

"On the other hand, if the symptoms do not begin soon after food, drink, or medicine has been taken (the circumstances being such as to exclude the possibility of poison being introduced by a wound, by the lungs, or by any other channel but the stomach,) the presumption on the whole is against poisoning, and sometimes the evidence to this effect may be decisive. The principle now propounded may beoften a very important one in the practice of medical jurisprudence; for when united with a little knowledge of the symptoms antecedent to death, it may be sufficient to decide the nature of the case. Thus it is sufficient, in my opinion, to decide the late celebrated case of the *Crown Prince of Sweden.* The Prince, while in the act of reviewing a body of troops on the 28th May, 1810, was observed suddenly to waver on his horse; and soon afterwards he fell off while at the gallop, was immediately found insensible by his staff, and expired in half an hour. As he was much beloved by the whole nation, a rumour arose that he had been poisoned; and the report took such firm root in the minds of all ranks, that a party of military, while escorting the body to Stockholm, were attacked near the city by the populace, and their commander, Marshall Fersen, murdered; and Dr. Rossi, the Prince's physician, after narrowly escaping the same fate, was in the end obliged to quit his native country. Now, no other poison but one of the most active narcotics could have caused such symptoms, and none of them could have proved so quickly fatal unless given in a large dose. It was proved, however, that on the day of his death the Prince had not taken any thing after he breakfasted; and an interval of nearly four hours elapsed after that till he fell from his horse. This fact alone, independently of the marks of apoplexy found in the head after death, and the warnings he had several times before it, was quite enough to show that he could not have died of poison, as it was incompatible with the known action of the only poisons which could cause the symptoms." 40.

We all know how often an outcry is raised against individuals or a family, when a member happens to die suddenly, the family itself not being in harmony with each other. In several instances of this kind, our author has been induced to dispense with an analysis, by resting on the criterion alluded to in the foregoing extract.

*Evidence from Morbid Appearances.* These, like the former, had great weight in evidence, but often with less reason. Except in the instance of a very few poisons, the morbid appearances *alone*, can never distinguish between the effects of them and of natural disease or violent death. Lividity of the body, and early putrefaction are still thought by the vulgar to be signs of poisoning; though there is no foundation for such an opinion. In poisoning by arsenic, it is well known, that there is the reverse of early putrefaction. The post-mortem appearances are inflammation of the intestinal canal and its products in one class of poisons—congestion of the cerebral vessels in another class—and in a third a combination of the two. But neither set of appearances are invariably caused by the poisons which usually induce them—congestion of the brain is rarely caused by those which are currently supposed to induce them and, in fine, most of the appearances of both kinds, are exactly similar to those left by many natural diseases. Still the post-mortem appearances are not to be neglected, as they will assist the investigator greatly, in connexion with other species of evidence.

*Evidence from Chemical Analysis.* This, of course, is the most decisive of all the branches of proof. It is esteemed most valid when the poison is detected in the stomach, intestines, or œsophagus—then in the matter vomited—and next, in articles of food, drink, or medicine of which the sufferer has partaken —and lastly in articles found in the possession of the prisoner. When poison is found in the stomach, a question may arise whether or not it was the cause of death.

We are next to inquire what are the causes which may remove the poison beyond the reach of the inspector—it may have been discharged by vomiting or purging—absorbed or decomposed.

In the trial of George Thom for poisoning the Mitchells, on the Aberdeen Circuit, 1821, it was clearly proved that the deceased had died from arsenic, although none could be detected in the stomach, for the man lived seven days, labouring under incessant vomiting. In a case where the patient lived only five hours, Dr. C. could not detect more than a fifteenth part of a grain of arsenic in the contents and coats of the stomach. A case is related in an American Journal, where a man had swallowed an ounce of arsenic, and died in eight hours—yet no poison could be detected by chemical analysis. And yet it is singular how ineffectual vomiting often proves in the expulsion of some poisons from the stomach. Arsenic and others which are not easy of solution may remain adhering to the villous coats, notwithstanding repeated and violent efforts to dislodge them by vomiting.

In respect to absorption, it has several times happened that laudanum, and even solid opium has soon disappeared in bodies poisoned with these substances. Dr. C. and Mr. Newbigging could not detect laudanum in the body of one who undoubtedly swallowed it, and who died in about eight hours. Pyl has related a similar instance. Great errors have been committed by medical witnesses in consequence of overlooking the fact of absorption. Dr. C. alludes to a recent instance which happened at a Coroner's Inquest in London.

"A young man one evening called his fellow-lodger to his bedside; assured him he had taken laudanum, and should be dead by the morrow; and desired him to carry his last farewell to his mother and his mistress. His companion thought he was shamming; but next morning the unfortunate youth was found in the agonies of death. The moral evidence was not very satisfactory; but that is of little consequence to my present object. The point in the case I would particularly refer to is the declaration of the medical inspector, that laudanum could not have been taken, because he did not find any by the smell or by chemical analysis in the contents of the stomach."* 52.

Lastly, the excess of a poison may be decomposed. Vegetable and animal substances may be altogether destroyed by the process of digestion. The case of a French soldier is related, who died in six hours and a half after swallowing two drachms of solid opium, none of which could be found after death, nor the smell of opium detected. Some mineral poisons, as sublimate, lunar caustic, hydrochlorate of tin, are also decomposed in the stomach, but they are not removed beyond the reach of chemical analysis. The decomposition is a chemical, not a vital process, and the base of the poison may be found in the solid contents of the stomach under some other compound form. The decomposition of the body also may render it impossible to detect poison that has been actually swallowed. Oxalic acid may be dissolved and then exude—vegetable narcotics may putrefy, and Prussic acid may be volatilized. Arsenic, however, has been detected in the body fourteen months after interment.

* "Morning Chronicle, Jan. 8, 1823."

*Experiments on Animals.* This is more equivocal than was once imagined; but our author thinks that some medical jurists have overstepped the proper limits in rejecting this species of proof in toto. Still it is not likely to be resorted to directly in cases of poisoning; but the practitioner should be acquainted with the opinions which have been given by others —too rashly no doubt—and which may be canvassed in a court of law. An important objection, indeed to this species of evidence, is the fact that what is poison to man is not always poison to animals, and vice versa. It is curious that the cat and the dog are affected by almost all poisons exactly in the same way as man—the dog more particularly.

"In general poisons act less violently on these animals; thus two drachms of opium are required to kill a middle-sized dog,* while thirty-six grains have killed a man, and probably a much less quantity would be sufficient for the purpose. It appears that one poison, alcohol, acts more powerfully on them than on man. There are also some poisons, such as opium, which, although deleterious to them as well as to man, nevertheless produce in general different symptoms. Yet the differences alluded to are not greater perhaps than exist between man and man in regard to the same substances; and therefore I think it may be assumed, that, on the whole, the effects of poisons on man differ little from those produced on the dog and cat." 55.

*Moral Evidence.* There is no doubt that the medical man, in the course of his professional investigation of the case, has excellent opportunities of acquiring information of the moral and circumstantial kind, which may often be of great advantage both to himself and the court. The heads of moral evidence, to which the medical practitioner ought to direct attention, while proceeding in his more immediately professional course, are the following.

"1. To suspicious conduct on the part of the prisoner before the event, such as dabbling with poisons when he has nothing to do with them in the way of his profession, conversing about them, or otherwise showing a knowledge of their properties not usual in his sphere of life:—2. To the purchase or possession of poison recently before the date of the alledged crime, and the procuring it under false pre-

* "*Charret,* in Revue Medicale, 1827, i. 514."

tences, such as for poisoning rats when there are none on his premises to poison, or for purposes to which it never is applied :—3. To the administration of poison, either in food, drink or medicine, or otherwise :—4. To the intent of the prisoner, such as the impossibility of his having administered the poison ignorantly, or by accident, or for beneficial purposes, alledged or not alledged :—5. To the fact that other members of the family, besides the deceased, have been similarly and simultaneously affected :—6. To suspicious conduct on the part of the prisoner during the illness of the person poisoned, —such as directly or indirectly preventing medical advice being procured, or the relations of the dying man being sent for, or showing an over-anxiety not to leave him alone with any other person, or attempting to remove or destroy articles of food or drink, or vomited matter which may have contained the poison, or expressing a foreknowledge of the probability of speedy death :—7. To suspicious conduct after the person's death, such as hastening the funeral, preventing or impending the inspection of the body, giving a false account of the previous illness, showing an acquaintance with the real or supposed effects of poison on the dead body :—8. To the personal circumstances and state of mind of the deceased, his death-bed declaration, and other particulars, especially such as tend to prove the impossibility or improbability of suicide :—9. To the existence of a motive or inducement on the part of the prisoner, such as his having a personal quarrel with the deceased, or hatred of him,—his succeeding to property by his death, or being relieved of a burden by it,—his knowing that the deceased was with child by him." 62.

*Imaginary, Pretended, and Imputed poisoning.* The leading points to be attended to in this kind of investigation are laid down by our author, and an actual example of each variety is given by way of illustration.

*Imaginary poisoning* can hardly occasion deception or embarrassment. The same hallucination which induces the belief of poison, will betray itself when the investigation bears upon the symptoms, the administration, &c. of the poison.

*Feigned or pretended poisoning* is much more apt to escape suspicion—and, when suspected, is difficult to develope satisfactorily, because the actor has it in his power to lay his plans with care, and even to make himself acquainted with the properties of the poison whose effects he intends to feign. Still he can rarely enact his part so well as to deceive a skilful physician.

"In a case of feigned poisoning an excellent mode of investigation is, after hearing out the individual's own story to put a number of questions involving an alternative answer, one alternative being compatible and the other incompatible with the alledged nature of his illness. No unprofessional person could stand such a system of interrogation, if skilfully pursued. Not only will his answers be often wrong; but likewise his manifest perplexity how to answer will of itself supply evidence of his falsehood." 77.

Great attention must also be paid to the chemical analysis. The following case will illustrate many of the rules to be observed on such occasions.

A remarkable trial for imputed poisoning occurred not many years ago, at the York assizes, where a man named Whalley was indicted for administering arsenic to Martha King, who was pregnant by him.

"The woman King swore, that the prisoner, after twice trying, but in vain, to prevail on her to take drugs for the purpose of procuring abortion, sent her a present of tarts, of which she ate one and a half,—that in half an hour she was seized with symptoms of poisoning with some irritant poison,—and that she continued ill for a long time after. Mr. Thackrah found arsenic in the tarts that remained untouched, and likewise in some matter that was vomited in his presence after the administration of an emetic, as well as in other matters of vomiting which were preserved for him between his first and second visits. Her appearance, however, did not correspond with the complaint shé made of her sufferings, her pulse and tongue were natural, and on careful investigation the following inconsistencies were farther detected. 1. She said she felt a coppery taste in the act of eating the tarts, a taste which arsenic certainly never possesses. 2. From the quantity of arsenic in the tarts which remained she could not have taken above ten grains while even after repeated attacks of vomiting, the alledged matter subsequently preserved contained nearly fifteen grains. 3. The first matters of vomiting contained only one grain, while the matter alledged to have been vomited subsequently contained fifteen grains. 4. The time at which these fifteen grains were alledged to have been vomited was not till between two and three hours after the symptoms began; in which case the symptoms would before that time have been violent. The prisoner was acquitted, and the prosecutor and another woman who corroborated her deposition afterwards admitted that they had entered into a conspiracy to impute the crime to him, because he had deserted her on finding that she was too intimate with other men." 80.

*Medico-Chirurgical Review.*

### 4. Dr. Monro on the Diagnosis of Hernia.

*Inguinal Hernia* We are all aware that males are peculiarly prone to this, and the other sex to the femoral hernia. The following tables, however, shew on a large and convincing scale the general laws respecting the individuals affected by the disease, the side of the body on which it is most trequent, and its more ordinary complications.

*From July* 6, 1805, *to July* 6, 1811. 1637 *Cases.*

| | | | Males. | Females. |
|---|---|---|---|---|
| 332 double | In both groins, | | 319 | 2 |
| | thighs, | | 0 | 11 |
| 1257 single | Inguinal, | Right side, | 671 | 26 |
| | | Left side, | 354 | 13 |
| | Femoral, | Right side, | 5 | 54 |
| | | Left side, | 2 | 37 |
| | In the naval, | | 13 | 76 |
| | Ventral herniæ, | | 3 | 3 |

Several patients having umbilical hernia, had also one or two inguinal or femoral herniæ, one person had a ventral, and two inguinal hernia.

The following document, extracted from the 25th volume of the Philosophical Magazine, corroborates the former. 3013 patients were examined.

| | | Males. | Females. |
|---|---|---|---|
| 741 Double Ruptures | In both thighs (femoral) | 3 | 44 |
| | In both groins (Inguinal) | 609 | 85 |
| 2272 Single Ruptures | In one thigh (femoral) | 57 | 136 |
| | In one groin (inguinal) | 1520 | 399 |
| | In the navel (umbilical) | 36 | 97 |
| | Total, | 2225 | 788–3013 |

Of the single ruptures, more than one third happened on the left side, and nearly two-thirds on the right side. A very small proportion of triple ruptures, and other extraordinary cases, likewise occurred in the above number ; but they were extremely rare, and mostly existed among the female sex.

These statements amply corroborate the general opinions regarding the sex most affected with inguinal or femoral hernia. They likewise prove the much greater prevalence of rupture on the right side than on the left, and the greater disposition in females to suffer from umbilical herniæ. Passing over the common varieties of the disease we stop at—

*Inguinal Herniæ, which in situation resemble Crural Herniæ, owing to Malconformation of the Inguinal Canal.*

The fourth modification of inguinal hernia is very rare, and occasioned by the malconformation of the inguinal canal, which is owing to the smaller tendinous fibres, which connect the larger columns of the external oblique muscle, being wanting.

In these circumstances the inguinal canal is imperfect; hence the bowels, when protruded, do not follow the course of the inguinal canal, but push immediately downwards and outwards, forming a tumour in the situation of a crural hernia.

This modification of hernia has been described by the late Professor HAMILTON of Glasgow, and also by PETIT. It remained for the late Mr. ALLAN BURNS of Glasgow to detect the cause of such a deviation, which he has described to me in the following letter, dated March 7, 1806:

The tumour was found in the bend of the left thigh. The inguinal canal was fully as large as it is usually met with in the male, and besides, so very short, that it presented, when fully unfolded, almost the appearance of a mere aperture. The round ligament of the womb was enveloped in a distinct tunica vaginalis, and bearing the same relation to the intestine that the spermatic cord does in the other sex. On the right side, the herniary sac was about two inches in length, and in shape resembling a Florence flask; the bulbous extremity extending from the lower orifice of the canal, was contained in the upper part of the thigh, *lying more in the course of a crural than of an inguinal hernia.*

This deviation from the usual direction of the tumour was produced by a premature separation from each of the external pillars of the inguinal canal. Where the inguinal canal is imperfectly formed, it is generally owing to the incomplete extension of the posterior or internal side of the ring.

Where this happens, the internal orifice of the canal is brought nearer to the pubes than it ought to be, but when the imperfection is produced by a premature separation at the external pillars, then, by dissection, we find the internal orifice in its proper place, but the external outlet is removed from the pubes.

In the first instance, when the herniary tumour protrudes, it lies just over the tubercles of the pubes, and follows the course of the spermatic cord into the scrotum, while in the latter it lies nearer to the spine of the ilium, and is seated just over the crural foramen, and by extension, descends along the thigh, *counterfeiting the appearance of femoral hernia.*

By attention, however, it is readily distinguished from the latter, by being felt lying over the crural arch, and on the outer side of the tubercle of the pubes.

When the bowels follow the course of the inguinal canal, the epigastric artery is situated nearer to the symphysis pubis than the hernia; whereas, when the bowels do not follow such a course, but pass only through the under abdominal aperture, then the epigastric artery is situated nearer to the anterior spinous process of the ilium than the hernia."

5. Excision of the Nerves in Cases of Neuralgia. By J. C. Warren, M. D.

As this operation has of late become rather unfashionable, we select three cases reported from the Mass. General Hospital in the Boston Med. and Surg. Journal.

Case 1.—*Excision of the Submaxillary Nerve.* Mr. S. aged 70, applied to me, stating that he had a dreadfully painful complaint in the face, and that he wished an operation for its relief. It appeared that he had been first affected without any obvious cause about fourteen years before. His pains were of two kinds ; first, a constant aching pain, which he compared to the worst toothache ; second, a spasmodic affection which occurred many times during the day. When he had the latter kind of attack, the muscles of the face quivered, the face became red and swelled, the eyes were filled with tears, and his intellect was for the moment suspended. For the last four years he had not been free from pain while awake, and his sleep was short and interrupted.

Respectable practitioners in the part of the country where he lived, had performed three operations, two on the sub-orbitar nerve and its branches, the third on the nerve of the lower jaw, where it comes out on the chin. These operations gave him a degree of relief, but the pain continued with a severity intolerable, and life had become a burden to him.

The pain he described as beginning near the ear, and thence darting into the lower and upper jaw, the lips, eye, forehead and scalp. The patient had made himself acquainted with the situation of the nerves of the face, and believed his pain to reside in the facial nerve, which he wished to have divided.

He was informed that an operation such as he wished might be executed ; but that in his case the affection appeared so general that there was no prospect of a cure ; and that, in fact, there were not any cases on record, of a successful division of the facial nerve at its root for this disorder. As soon as the patient understood that the operation was practicable he desired to have it performed, and agreed to enter the Hospital, on account of the superior advantages it afforded over any private situation.

The operation was thus conducted. An incision two inches long was carried from the back of the ear downwards in front of the mastoid process. The edge of the parotid gland was then exposed on one side, and on the other, the anterior edge of the mastoid muscle. The dissection being continued between the parotid gland and the mastoid process, the facial nerve was exposed where it crosses this space and passes on the gland, it was divided and a portion cut out. When the nerve was cut, the muscles of the face quivered and were paralyzed ; but the patient said he merely perceived the division ; that it was not attended with an acute pain, and that the principal cause of his sufferings was not reached.

After this operation, it appeared that the pain in the upper part of the face was diminished ; perhaps removed. But the patient now became sensible that the most acute pain, and that which probably had existed first, was seated deeply in the lower jaw, beginning at the zygomatic arch and shooting into the bone. It was entirely independent of the wound made in the operation. From this wound he experienced no inconvenience, was unwilling to speak of it, and scarcely wished it dressed, so greatly was he disappointed at not being relieved from his sufferings. He begged his case might be again taken into consideration, and something more if possible devised for his relief. A meeting of the consulting physicians and surgeons being called, I proposed the operation which is described hereafter, and it being agreed to, was performed nine days after the first.

An incision was made over the side of the jaw, from the semilunar notch to the inferior edge of the bone. The parotid gland being exposed, was divided as far back as possible, and turned forwards. Then the masseter muscle was divided in the course of its fibres to the bone, and afterwards the edge of the knife being turned forwards, some of the fibres were transversely cut, in order to make room over the bone. A trephine, three quarters of an inch in diameter, was then applied half an inch below the semilunar notch, midway between the anterior and posterior edges of the jaw ; and the circular piece sawed through and removed in two parts, the external table by a lever, the internal by forceps. Between these pieces lay the nerve, with its accompanying artery and vein, at the point where they penetrate the bone. At the superior edge of the hole in the bone was seen the large internal maxillary vein, pulsating from the movements of the artery. The sub-maxillary nerve being now raised on a probe, the patient directly exclaimed that this was the seat of his sufferings. Half an inch of this nerve was cut out, and on examination, it was found to comprehend the branch given to the internal face of the lower jaw. The artery was tied without difficulty. The transverse artery of the face had been previously tied on each side of the wound. A suture was employed to bring together the two parts of the parotid gland, and the wound closed by adhesive plaster.

The patient said that the pain of this operation could not be compared with that from his disorder. The pain on the wounding and dividing the maxillary nerve was most acute.

On the evening of the operation he was relieved from pain, the first time for four years ; and had no return of it afterward. On passing a probe into the wound three or four days after, a branch of nerve in the masseter was touched, and a violent pain produced. The pain did not, however, return, nor had he any threatening of his former attacks. On the nineteenth day from the second operation, his wounds being nearly healed, he left the Hospital perfectly cured of his disease, with the strongest conviction that it would not recur, and not a little gratified by his own perseverance.

Case 2.—*Excision of the Infra-Orbitar Nerve.* Encouraged by the result of the last case, I operated in the same manner in the following cases.

*Mary Tucker, aged fifty-one, admitted into the Mass. Gen. Hospital 24th April,* 1824.

Has a pain in the left side of the face, situated in the infra-orbitary foramen, but often extending to the jaws and occasioning a sense of tightness and burning in these parts. She describes the pain as acute and lancinating, and as always aggravated by exposure to cold and by fatigue. During the paroxysms her vision is much impaired, and when they are severe, the face swells very much. There are moments when she is free from complaint, but talking or exertion is apt to bring it on again. The disease is most troublesome at night, and prevents sleep. Says she has not slept all night for a number of years.

She has had this disease for eighteen years. For six months past has had almost constant pain. She says she has had the cow-pock, was very ill, and in her recovery had pain and stiffness in the left side of her face, with an inclination of the face to the left side. This lasted three months; during the warm weather she was free from this affection, but, in cold weather, it recurred, and has gradually assumed its present form. She had rather a feeble constitution originally, but enjoyed tolerable health previous to the present disease. Has occasionally used anodynes with advantage at night. Has always been rather costive.

R. Solut. Sulph. Magnes. ℥ij.

29th.—*Operation.* The head was inclined to the right shoulder, resting upon the breast of an assistant. An incision was then made directly over the nerve, from the orbit, downwards about an inch. The seat of the nerve was made certain by passing a probe into the wound. Then the nerve being fully exposed, was divided near the infra-orbitar foramen; a piece half an inch long was dissected out and removed with its incipent ramifications. After the operation, a few small vessels bled considerably, which were restrained by sponges and cold water. The patient was then returned to her bed.

30th. At two o'clock, yesterday, the sponge was removed, and the vessels bled as copiously as before. A layer of lint was placed in the wound. The patient complained of some soreness and pain last evening, and was directed to take thirty drops of laudanum. This morning is tolerably comfortable; says she passed a quiet night. Now the situation of the pain has altered; since the operation, it is more confined to the parts about the lower jaw. Bowels well. Face a little swollen. From this time the pain gradually diminished, and in June she left the Hospital, entirely cured.

Case 3.—*Margaret Andrews, widow, aged* 57. *Entered the Mass. Gen Hospital March* 16, 1827.

Six years ago had a carious tooth in upper jaw, on left side: at the same time began to have darting or jumping pain on left side, commencing at this tooth and passing up the cheek. Had the tooth extracted with some relief. Paroxysms of pain have gradually increased in violence and duration—are worst, as she thinks, in August and September. They now commence as at first. The pain passes to the cheek-bone where it is most severe : it then starts in two courses, one passing the external, the other the internal angle of the left eye, and is again felt at a spot on the forehead. The pains sometimes extend to the back of the head. When the pain is most severe, she cannot bear the slightest touch upon the cheek. Severe paroxysms produce nausea ; they are brought on by taking hot or cold substances, or by exposure to cold air. Her appetite is tolerably good ; tongue clean,—is unable to extend it equally on both sides. Bowels prone to costiveness. Ceased menstruating at forty-eight or forty-nine ; does not know which. Has used pills of extract of stramonium and ipecac, with some relief.

The extract of stramonium, the carbonate of iron in large doses, the extract of belladonna with blisters having been successively tried for more than a month without benefit, the operation was determined upon, and performed in the following manner.

An incision one inch and a half long was made from a point half an inch below the middle of the orbit towards the middle of the left half of the upper lip ; the periosteum was exposed to view with the sub-orbitar nerve lying thereon and beginning to divide. The nerve, as it arises from the sub-orbitar foramen, was cut across, then the inferior part being raised and dissected up, for half an inch, was cut off. The patient expressed some pain on the touching and division of the nerve, and at the same time a satisfaction at feeling that the source of her suffering was reached.

After the operation the tics never appeared ; but she had some pain, which rapidly diminished till the 14th of the following month, when she was discharged : and I had the satisfaction to hear from her some months after, that she had no pain subsequently to her quitting the hospital.

6. *Cæsarian Operation on a Patient dead from Hæmoptysis.* In the Hospital St. Louis.

Eliza Doviller, æt. 28, entered the Hospital St. Louis on the 2d of June, 1829, with the usual symptoms of phthisis pulmonalis ; cough, blood-streaked expectoration, sharp pains in the chest, hectic fever, emaciation.

We need not particularize the treatment pursued in the hospital, suffice it to say that on the 14th, the weather being hot and oppressive at the time, she suffered much from suffocation and spat more blood than usual. A small bleeding was had recourse to and procured some relief, but at 7 P. M. of the 17th she was suddenly seized with a frightful hæmoptysis, and expired in the space of four minutes, with the blood gushing from the mouth and nostrils.

When the interne, M. Huguier, arrived, life was quite extinct, and he instantly determined to lay open the uterus and save the life of the child if possible. The corpse being placed with the head and chest raised by pillows and the thighs flexed upon the pelvis, an assistant retained the uterus in the middle of the abdomen. The integuments then being put upon the stretch by the left hand applied below the umbilicus, a vertical incision was made with a convex bistoury along the median line, commencing at an inch below the umbilicus and terminating at an inch and a half or two inches from the pubes. The skin, cellular tissue, linea alba, and peritoneum were all cut through, with a vast deal of superfluous caution considering that the patient was a dead one, and at length the anterior and superior portion of the uterus was incised "layer by layer," till the membranes were exposed, punctured, and the liquor amnii evacuated. The fore and middle fingers of the left hand were introduced into the opening and the latter enlarged by tearing the membranes, when the whole hand was got into the cavity of the womb, and the sides of the incision separated, by the fingers being held wide apart at its superior angle.

The feet of the child were now sought for with the right hand and quickly found, when both hands were withdrawn and employed to extract the infant which scarcely gave any signs of life. It was pale, exsanguined, motionless, and the pulsations of the heart were scarcely perceptible. The cord was tied before it was cut, frictions with warm cloths on the præcordia were had recourse to, artificial respiration set up, and the child placed in the warm bath. Under the employment of these measures the heart began to beat more vigorously, the nisus of respiration was induced, and the infant uttered that feeble cry which heralds our entrance into a stormy existence. The animal heat could only be retained by keeping the little creature in warm cloths, but at the age of thirty days, when the report closes, it enjoyed good health.

No dissection of the unfortunate mother was permitted by the friends, but that does not matter very much, as the rescue of the offspring by the Cæsarian section is the important portion of the case. Whether an existence so procured will be one of happiness or misery to the infant, whether it bears in its organization the germs of its future destruction by scrofula or phthisis, or whether it does not, it must at least be allowed that no case can possibly arise, in which the Cæsarian operation can inflict a less amount of suffering, in proportion to its good, than it has done in the present instance. The knife of the surgeon or the anatomist "wrings not the tethers" of the dead!

7. *Diagnosis of Gastric and Intestinal Inflammation by inspection of the features of the face.* The Hospital for Children, (Paris), is appropriated to the reception of children of both sexes under the age of 16 years, whatever may be the nature of their complaints. Messrs. Jadelot and Guersent are the medical officers, and, in their lectures, make known to their students, the results of their extensive experience.

M. Jadelot was among the first to acquire an accurate knowledge of gastric and intestinal inflammations; he created for himself, so to speak, new methods of recognising them, and arrived, by the inspection of the features of the face alone, at an accuracy of diagnosis truly remarkable. This *semeiology*, peculiar to early life, though we can sometimes derive advantages from it in more advanced years, M. Jadelot has named physiognomonic : in a very few words, a succinct idea of it may be afforded to the reader. Independently of the alterations of colour which the face presents, and which furnish to the physician signs more or less positive, the expression of the physiognomy, and the prominence, more or less considerable, of particular traits, are also means of clearing still farther the diagnosis.

In the infant, whose facial muscles are not endowed with great mobility, three principal traits present themselves to the observer. They are nearly parallel, and direct themselves from the centre towards the sides of the face ; the first, (*oculo-zygomatic*,) proceeding from the great angle of the eye, loses itself a little below the projection formed by the cheek-bone ; the second, (*nasal*,) commences at the superior part of the wing of the nose, and prolongs itself, in a semicircle, towards the commissure of the lips ; it is sometimes crossed by a small trait (*genal*) which directs itself towards the middle of the cheek ; the last, (*labial*,) arises at the commissure of the lips, and loses itself towards the chin.

Each of these traits is in relation to one of the visceral cavaties. The first is connected with the diseases of the cerebro-nervous system ; the second, and its accessory, (the *genal*,) with abdominal lesions ; the third, lastly, indicates the affections of the organs of circulation and respiration.

This mode of investigation, valuable in regard to patients incapable of furnishing any clear information to the physician, requires much experience ; but the results obtained from it indemnify the physician for the trouble to which he submits, in order to gain his object.

8. *Lupus treated by the Iodide of Mercury.* One very interesting case of this disease was in the Hospital (Glasgow Royal Infirmary) for some months. I mention it here, because it has been greatly benefitted by a medicine which is not yet much employed in this quarter ; I mean the iodine of mercury.

A. A. a healthy country girl, aged 21, had suffered under lupus for thirteen months at the time she was admitted. The tip and alæ of the nose were the parts affected, and there was a small portion of the cheek also involved, and the disease had in some degree spread to the inside of the nostrils. It presented in several places the distinct tuberculated appearance of lupus, and in other places had proceeded to ulceration, although this was not very deep. She had undergone a course of mercury, and used a great many local applications ; among which were the nitrate of silver and a solution of arsenic, but with little permanent benefit. After her admission lunar caustic was repeatedly applied to the affected parts, and she used an ointment composed of mercury, camphor, and turpentine, and also a strong arsenical ointment, but not with much effect. The iodide of mercury, in the proportion of six grains and afterwards nine grains, to an ounce of simple ointment, was then used daily, and under this application many parts skinned over, and the disease was in a great measure subdued. She used it for several weeks in the Hospital, and since she was dismissed some of the small tubercles have re appeared, but were speedily destroyed by the slight ulceration produced by the ointment. In this case, the disease, although not yet completely eradicated, has been kept under, and more completely relieved by this, than by any other application.

I have also used the same preparation, but stronger, in a case of curious anomalous tumours of the face, somewhat resembling the melanosis, but evidently not malignant, although it could not prevent the re-appearance of the affection. I think this preparation of iodine and mercury will be found to answer in many cases of ulcerations where other escharotics, murias hydrarg. nitras argenti, arsenic, &c. have failed."

Here we must stop for the present, but some interesting cases remain to be noticed at a future opportunity.—*Med. Chi. Review.*

9. *Case of Cystocele which was not recognized for a great number of years ;* recorded by M. Ribell of Perpignan in the Archives Generales. The patient fifty-eight years of age, had suffered since birth from a large inguinal hernia in the left side, and since the age of fifteen from a smaller one on the right. A truss was not used until the age of 25, when one was applied which made ineffectual compression. The difficulty of making water began about the age of 28. Five years after he applied to several surgeons at Perpignan, who informed him that he had strictures, and treated him accordingly.

On the 22d of August, 1828, having been troubled for five days with more than ordinary dysuria, M. P*** was suddenly seized in the evening with complete retention of urine. After much straining he voided a little pure blood, and subsequently some blood mixed with a little urine and much mucus. Our author was summoned from Perpignan, but owing to the distance was unable to arrive until after the lapse of 24 hours. He had then just made water, though with difficulty, and was free from any fever or excitement. On a subsequent visit our author determined to satisfy himself of the state of the bladder and urethra, but on introducing an instrument, he was not a little surprised to find that it could be passed without difficulty or obstruction of any description, and half a wine-glass full of limpid urine evacuated. It was quite clear from this fact that there was not, and never had been a stricture of the urethra. But what was there then to account for the dysuria of thirty years duration ? By dint of close inquiry and accurate examination the following particulars were ascertained.

1mo. The difficulty of making water was always greatest when the inguinal hernia on the left side was most voluminous.

2ndo If the nisus of micturition occurred at the moment when the hernia was small, the urine escaped in a free stream, but if during its evacuation the hernia escaped or enlarged, the flow of the urine was abruptly checked.

3tio. Experience had taught the patient, and without his knowing how or why, that in order to make water easily, he should raise the hernia and make the neck the most dependent part.

4to. After making the patient retain his water as long as possible (scarcely four hours) and keeping him on his legs in order that the hernia might protrude to the utmost, the catheter was introduced and about four spoonfuls of water only abstracted ; this too could not be effected till the hernia had been reduced.

5to. A forcible injection of two wine-glass-fuls of water into the bladder produced the following results :—It excited a very strong desire to make water, the patient not being accustomed to retain so much liquid in the bladder at a time : it produced a very sensible enlargement of the inguinal hernia though the patient continued recumbent, and it occasioned a distinct sense of fluctuation in the hernial tumour towards its neck.

6to. The hernia on the right side (an intestino-omental one) was readily and completely reducible.

7mo. The left, on the contrary, was irreducible. It appeared to contain intestine towards its lower part, and the testicle seemed to be united to the gut. The neck of the tumour, when grasped between the finger and thumb, gave the sensation of comprising within it two lubricated membranes slipping on each other.

From the foregoing premises, M. Ribell concludes that his patient is affected with a congenital hernia on the left side, originally intestinal ; that this hernia not being restrained by any pressure has insensibly enlarged, and involved the bladder ; and finally that the latter viscus has contracted adhesions in its new situation, and is consequently irreducible.—*Ibid.*

10. *The lower orifice of the nasal canal.* Below the anterior part of the os spongiosum inferius, and under the meatus inferior, we find this opening is generally elliptical, oblique in its direction, and about six lines distant from the beginning of the nares : it is concealed under the os spongiosum inferius, and turned a little backwards : its breadth varies considerably. The nasal canal proceeds obliquely upwards, forwards, and a little outward, towards the great angle of the eye. It is sometimes situated entirely in an excavation of the superior maxillary ; but, generally, the os spongiosum inferius and os unguis equally contribute to its formation. The membrane which lines it is a continuation of the pituitary ; and it sometimes forms a soft and pendent fold of a greater or less length, which is situated against the external wall of the canal. It is by this opening that, according to Laforest, sounds are introduced into the nasal canal, to re-establish the flow of tears in the case of fistula lacrymalis. In order to reach this place, the sound must glide on the floor of the nasal fossa, under the inferior spongy bone, by turning the convex side of the instrument towards the septum, to the place where we feel that its point has passed beyond the ascending branch of the superior maxillary. Then the sound is turned between the fingers, in order to direct the point above and within the side of the eye, taking care to make it glide, during this movement, on the wall of the lower meatus. The handle of the instrument is thus depressed by this rotatory motion, its point being placed in the nasal canal, and then made gently to advance towards the lacrymal sac. This operation, generally easy to perform on the dead, is very difficult on the living subject, not only on account of insupportable tickling which it occasions, but also numerous variations which are met with in the anatomical disposition of these parts. Indeed, this opening is sometimes so narrow, that it admits with difficulty a very small probe ; or the pituitary membrane which lines the nasal canal extends beyond it, and forms a kind of curtain round this opening. In some cases, the partition of the nasal fossæ is inclined so as to press against the lower spongy bone ; in others, this eminence is placed so low, that the probe passes on its superior surface, whatever may be the effort made to cause it to glide beneath ; or rather its free edge is prolonged, so as to transform the lower meatus into a kind of canal For these reasons the operation has been abandoned : moreover, inflammation of the pituitary membrane, and fracture of the lower spongy bone have sometimes occurred from the attempts to perform the operation.

*Edwards Manual of Surg. Anatomy.*

11. *Spontaneous Combustion.* Professor Dupuytren says, that many authors have attributed the accident of spontaneous combustion to a combination of alcohol with the living solids ; and this opinion they maintain without regard to the origin of the combustion, whether commencing primarily in the individual, or communicated to him from without.

M. Dupuytren, without denying that most of the poor subjects of this terrible phenomenon are given to hard drinking, formally denies that the combination of alcohol with the tissues is the cause of the burning.

M. Dupuytren seems to be addressing his class. "It has been denied," says he, "that any other than a spontaneous combustion could consume a whole body in a single night. I, like you, passed my youth in dissecting rooms, aud perhaps more than any of you, I consumed my time in dissections ; but that was thirty years ago, and subjects did not then abound as they now do ; they were very difficult to get, we were obliged to rob the cemeteries, and it was still more difficult to get back the debris. It was thought better to get rid of them in another way, and we burnt them. For this purpose faggots were piled up in the chimney, and the remains of two, three or four bodies placed upon them ; a fire was made in the evening, and next day the whole was found to be consamed. We also took care to place the fattest pieces lowest, and the combustion was more rapid, more complete in proportion as there was a greater quantity of fat. But one night was always long enough for the entire combustion."

M. Dupuytren applies this practical observation to the consideration of spontaneous combustions. He knows of no instance of it in a lean dry individual : every one of the cases, without exception, occurred in extremely fat people.

If it be observed that the chamber in which a spontaneous combustion has taken place is found full of thick vapour, the walls covered with black carbonised matters ; that there are commonly observed streams of grease on the floor, with some ashes, and occasionally fragments of bones, the sole remnants of a body shortly before organised, our remark will obtain additional credence. The above is an account of what is observed : here follows an explanation ; and this is the way in which the facts most commonly take place.

A woman (it happens mostly to women) returns to her house after having taken a pretty strong dose of spirituous liquor. It is cold, and a fire is kindled ; she seats herself on a chair, with a foot stove under her feet. To the coma produced by the spirituous liquors is added the asphyxia occasioned by the charcoal ; the fire is communicated to the clothing ; her insensibility is too great to permit her to feel pain ; the clothes are consumed ; the skin burns ; the epidermis cracks open, and the fat melts and flows out ; a part runs on the floor, and the rest serves to support the combustion ; day comes at last and the whole is consumed.

This is the way that alcohol has been the occasional cause of combustion ; it is by producing coma, and not by a pretended amalgamation with our tissues, that it acts upon the human body in this striking manner.

Dr. D. cites the following reasons to show that combustion is never purely spontaneous :

In dissecting rooms it is common in warm weather to find the bodies of half putrid subjects surrounded with a phosphorescent light ; but such subjects are never consumed.

A wretched woman, who was excessively fat, returned late one evening to her house, after having drank freely of ardent spirits. It was cold weather, and she seated herself on a chair in the middle of the room. In the morning a thick smoke was observed to fill the whole chamber ; streams of grease were observed on the floor, the chafing dish, some remnants of the chair, and a small quantity of ashes, were all that were found.—*N. Amer. Med. & Surg. Journal.*

12. *Tapping in Hydrocephalus.* The following case is extracted from the number of "the London Medical and Physical Journal" for May last. The subject was presented by Dr. Conquest to his class, at St. Bartholomew's Hospital.

"A girl of about two years of age had several signs of hydrocephalus, from a date soon after its birth, and for many months past the head had gradually increased, until it acquired an enormous size. The forehead was singularly broad, and the anterior fontanelle unusually large. The pupils were permanently dilated ; the child slept almost incessantly, and frequently had two or three frightful convulsions during the day and night. Dr. Conquest operated some time since, before a large number of the pupils of the Hospital, by pushing a very beautifully constructed trocar into the right lateral ventricle. He introduced it obliquely, close to the edge of the right temporal bone, about midway between the crista galli process of the ethmoid bone and the anterior fontanelle, so as to avoid the longitudinal sinus on the one hand, and the corpus striatum on the other. The instrument entered about two inches below the scalp. An ounce and a half of bloody serum, mixed with portions of the cerebrum, escaped. The pulse became feeble, and temporary collapse followed. The fluid was allowed to escape stillicidium ; and within eight and forty hours, about two pints and a half flowed out of the opening. Almost immediately after the operation, the pupils became sensible to the stimulus of light : the drowsiness was succeeded by disinclination to sleep ; and the pulse, which had always before been remarkably slow, became about eighty. Two days after the operation, the brain evidenced signs of inflammation, with high constitutional disturbance ; and great alarm was excited by a rather formidable attack of convulsions. Leeches to the temples, and the constant application of cold to the head, subdued the local inflammation, and within twenty four hours all became tranquil. The head was well strapped, and from the cessation of cerebral excitement, no unfavourable circumstance occurred."

When this child was exhibited, every one was struck with the improvement of its appearance, and by the intelligence and cheerfulness of its countenance. Dr. C. stated that he considered the child perfectly well, and "as exhibiting a most gratifying and triumphant proof, that this seemingly formidable procedure might be safely and successfully adopted under similar circumstances."—*Ibid.*

13. *On the use of Salicia in the Treatment of Intermittent Fever.* Our readers are perhaps aware, and if they are not, we inform them, that salicia is a substance not long since discovered in the bark of the poplar—salix alba, by M. Leroux, and an apothecary of Vitry le Franqais in France. M. Miquel was led from a large number of experiments to conclude, that this substance is equal, as a febrifuge, to the sulphate of quina. He has published in the Gazette Medicale, nine cases of intermittent fevers in which this remedy was employed. In eight the success was complete. In two, the disease was so mild, that it would probably have yielded readily without the use of any febrifuge. Some other cases were examples of the most simple form of intermittent fevers, which are usually with success managed by means of the sulphate of quinia. They were only cured after the salicia had been employed from two to five days. In one case, however, the disease was of long continuance, and had resisted the use of the Peruvian bark, opium, assafœtida, &c. In this case the salicia was completely successful. In the ninth case, the remedy failed ; but it is to be observed, at the same time, that in this instance, the sulphate of quinia, in like manner, failed to render the desired effect.

The salicia is administered in larger doses than the quinia—from 18 to 20 grs. the first day—afterwards from 30 to 40 or even 50 grs. At whatever dose it is used, it does not give rise, as the sulphate of quinia sometimes does, to a burning sensation in the stomach, a circumstance which will cause it to be preferred to the latter in individuals with irritable stomach.—*Archives Generales, for January, 1830.—Ibid.*

14. *Treatment of Puerperal Mania.* In a letter addressed to the Editor of the London Medical and Surgical Journal, and inserted in the March number of that work, Dr. BLAKE, of Ipswich, sets down true puerperal mania as a disease very nearly allied in its nature to delirium tremens, "although it does not arise from precisely the same causes," nor observe exactly the same paroxysmal form.

"It is a well known fact," he remarks, "that during the greater part of the period of utero-gestation, the vascular system is in a highly plethoric state, and the blood itself exhibits, when drawn, properties similar to those which exist in it during the height of inflammation ;* now it must seem evident that such a condition, existing for so many months in the animal economy, cannot fail to act, through the medium of the blood-vessels, as a strong and continued stimulus to the brain and nerves ; it therefore ought not, in my mind, to appear surprising, that in peculiar states of the female constitution, mental alienation, or puerperal mania, should be the consequence of the sudden privation of such powerful and long accustomed stimulation, which is the natural result of parturition and its consequences. The brain and nervous system in this case suffer, subsequent to delivery, from the loss they have sustained by the sudden privation of that stimulus which was afforded to them during gestation, by the distension of the blood-vessels, and the stimulating nature of their contents, in the same way as I have explained delirium tremens† to supervene in subjects accustomed to the stimulus of ardent spirits, &c. &c. on the sudden cessation of their habitual intemperance. Like delirium tremens, also, this disease does not come immediately on the occurrence of the cause, but takes a given time, according to the nature of the constitution, and the degree of previous stimulation, before it becomes developed, and extreme debility in both cases is generally observed to precede the stages of delirium ; in short, puerperal mania seems to me to run a course, though generally much more protracted than that of delirium tremens, which would entitle both disorders to be arranged in the same class, order, and genus."

---

* Vide Edinburgh Medical and Surgical Journal for Oct. 1823, or the London Medical and Physical Journal for Nov. of the same year.

† See a paper on the buffy coat of the blood, in the Medical and Physical Journal for November, 1829.

As regards the treatment, he says, "Our object should, of course, be to endeavour to induce such a state of the system as would resemble, in the greatest possible degree, that which existed previous to delivery. To effect this, the horizontal position should be carefully observed, in order to favour the ascent of the blood to the head; on the same principle the abdomen should be properly supported by a roller, and all drains on the system, such as giving suck, &c. should be particularly avoided. The various secretions should at the same time be carefully regulated, and local determinations relieved on general principles; during all this the strength ought to be supported by a light but nutritious diet, together with a moderate administration of diffusible stimuli, joined to sedatives, antispasmodics, and tonics, as the peculiarities of each case might indicate. These, with good air and the usual attentions, both moral and physical, which are found advantageous in the guidance of insane patients, are, apparently, the means most likely to prove efficacious in this often times very distressing nervous delirium; and on reference to the works of Denman, Professor Burns, Drs. Gooch, Marshall, Hall, Abercrombie, Ryan, and others, they will not be found to differ essentially in principle from the *methodus medendi* proposed by these authors, although they have not taken the same view of the causes of the disorder that I have ventured to do."

He suggests the propriety of resorting, in extreme cases, to transfusion.—*Ibid.*

15. *Excision of the Knee Joint.* This has been performed by Mr. Parks, of Liverpool, and Mr. Crampton, of Dublin, with success. Mr. Syme has followed the example. His first case was John Arnot, aged eight years, whose left knee was much enlarged, and immovably bent at an acute angle with the thigh, with two sinuses on the inner side of the joint, which allowed a probe to reach the bone. Excision was performed. No dangerous irritation resulted, but much difficulty occurred from the contraction of the flexor muscles preventing the suitable apposition of the sections of the leg and thigh bone. This was gradually overcome by continued extensions and counter-extension. "Four weeks after the operation, the wound was all but healed, and the limb is now becoming every day more useful to the patient, who can already make considerable use of it in walking, though not yet provided with a high-heeled shoe."

In a second case, similar to the above, in a female aged seven years, excision was performed. The same difficulty wrs experienced from a contraction of the ham-strings, which could not be effectually obviated. In this case it was found that the os femoris was extensively denuded and diseased. The patient's strength became exhausted, and she died on the 8th of January. The operation was performed on the 28th of December.

16. *Premature Labour.* Dr. Caepbell gives a case of premature labour artificially excited, in the Edinburgh Medical and Surgical

Journal for April last. The fœtus was born alive, but afterwards perished from convulsions, in consequence of the injury received during birth. Dr. C. is a decided advocate for inducing premature labour in cases of moderate deformity of the superior strait; but contends that the child's safety is much endangered by puncturing the membranes and allowing the liquor amnii to escape. He says, it is merely necessary, by means of the finger, or a small catheter, *to separate the membranes from the uterus for a short distance.* Labour usually supervenes in from three to seven days; the patient should walk about, and occasionally take a cathartic. She should avoid straining, so that the membranes need not be ruptured, if possible, until the head is about to make its exit from the vagina. In this way the child suffers less from pressure.

17. When M. Wœlher, in 1828, published his method for obtaining the metal of alumina, by the decomposition of the chloride of aluminium by potassium, M. Bussy supposed from analogy, that glucinium might be obtained in a similar manner from the chloride of glucinium. Experiment showed the correctness of this opinion. Since then, he has been successful in insulating magnesium from the chloride of magnesium.

*Preparation of the chloride of magnesium.* Though magnesia at a red heat may be decomposed by chlorine, yet the chloride is obtained with difficulty in this way. It may, however, be obtained readily by previously mixing the magnesia with finely divided charcoal. For this purpose, M. Bussy mixes together equal parts of calcined magnesia and starch, by the aid of a little water, and divides the whole into small pieces, which are strongly calcined in a covered crucible. The pieces are then placed in a tube of porcelain, through which a current of chlorine is passed, and the tube heated to redness. After some time the chloride of magnesium runs along the tube, and solidifies at its extremity. It is in the form of a white crystalline mass, exhibiting, when broken, large, brilliant laminæ, slightly flexible, and having the aspect of spermaceti. It is very soluble in water, and of a sharp and bitter taste. When exposed to the air it attracts moisture very rapidly.

*Preparation of the magnesium.* A glass tube is selected, rather strong, about the third of an inch in internal diameter, and fifteen or twenty inches long, and bent at one end in the form of a retort. After having introduced five or six pieces of potassium, about the size of a pea, into the bent as well as the straight part of the tube, fragments of the chloride of magnesium are placed in it, separated from each other by bits of porcelain, to prevent them on fusion from running together into a mass. This portion of the tube is then heated, and when it has nearly attained a dull red heat, the potassium is made to pass in a state of vapour, by heating the bent part of the tube. A strong incandesence immediately occurs, which extends gradually throughout the whole tube. When the tube has cooled, the matter within it presents white metallic globules, disseminated in the undecomposed chloride. When thrown into water, some hydrogen is disengaged, due to the presence of some potassium, and magnesia precipitated from the action of the potassa on the chloride, and brilliant globules, having the lustre and whiteness of silver, fall to the bottom of the vessel.

*Properties of magnesium.* White like silver. Very brilliant and very malleable. Fusible at a moderate temperature. Unalterable in dry air, but losing its metallic lustre in a moist atmosphere, in consequence of the formation of a superficial layer of oxide. When burnt in minute portions, it scintillates like iron in oxygen, but larger pieces are converted slowly and with difficulty into pure magnesia. Pure water deprived of air, has no action on it, unless when boiling, when a few bubbles of hydrogen are disengaged. Certain saline substances promote remarkably the decomposition of water by magnesium. Dilute acids dissolve it with disengagement of hydrogen. It unites with mercury, but not without the aid of heat, and destroys the fluidity of that metal even when present in very small quantity.
*Ibid,*

